# Some Observations on the Use of Apomorphine

**Published:** 1912-07

**Authors:** J. S. Horsley

**Affiliations:** West Point, Ga.


### SOME OBSERVATIONS ON THE USE OF APO-MORPHINE.

By Dr. J. S. Horsley, West Point, Ga.

(*Copied from the "Medical Record," Dec.* 6th, 1890.)

In presenting this subject for the consideration of this Association, I do so with a sense of modesty growing from the knowledge that there are those present who perhaps have had ampler scientific methods at hand for a study and a greater variety of phenomena upon which to demonstrate its use. The chemical formula of this medicine need not enter here to consume your time, as it is the practical results of its use with which we are more personally concerned.

In 1890 a short article was published for me in the New York Medical Record of December 6th, "Some Observations on the Use of Apo-morphine," which received quite an extended notice, as this was among the first notices published, bearing on this particular phase of its action, viz., its prompt control of motor disturbances, however produced. One case, illustrating its use in poisoning by strychnine, showed that it probably prevented the death of the patient by asphyxia; this apparently being threatened with every spasm, which was of frequent and increased severity. Other cases, illustrating its use in sick headache, of such character as to threaten general convulsions, palpitation of the heart with great dyspnoea—effects of excessive drinking, etc. I present these cases for your consideration as probably many of you did not have a chance to read this report when first made. I then presented this remedy as one of great value in controlling motor disturbances, whatever the cause. I am presenting the remedy to-day with greater assurance of its use than I had at that time, in a greater variety of suffering than any one remedy within my knowledge. Hysteria, in its varied forms, quickly subsides with 1-40th grain given subcutaneously. The raving maniac from drink quietly sleeps off his delirium. Spasms of children are quickly relieved by 1-80th to 1-60th grain. I will not take your time by further specifying, but say that when you have a motor disturbance present or threatening, apo-morphine in proper dosage is the indicated remedy.

I would like to impress the general practitioner with the importance of apo-morphine in prevention of puerperal eclampsia given long enough before the attack would occur. I do not say that this is an infallible remedy in such cases, but given timely, it will prevent the attack and in a large majority of cases stop the attack when present; this due to the exciting cause of eclampsia being an irritation of the stomach by its contents of undigested food, bile or anything which under normal conditions should provoke vomiting. The eclamptic seizure being a general convulsion, determined by distribution of the impulse, which should have been received by the vomiting center alone.

A very great number of special cases could be given showing its use, but I will not take your time with further detailed cases except to mention that sufferers from asthma, capillary bronchitis (in selected cases) are promptly relieved by 1-40th to 1-20th grain. The danger attending morphine habit, which so often attends cases of asthma treatment, is obviated by apo-morphine, and yet gives more prompt and perfect relief than morphine. I would say further that since apo-morphine is a depressant to the motor centres, and over-stimulation of these centres is a condition which we wish to allay, that strychnine should not be given conjointly with it; as a larger dose of apo-morphine would be required to produce the desired effect. However, if an overdose of apo-morphine has been taken, we would expect strychnine to meet the condition. Theoretically, apo-morphine and strychnine should antidote each other. It goes without saying that great care should be taken in administering it to the very weak or very young.

Case 1.—In August, 1889, Mrs. N., aged 22, mother of four children, had a very severe attack of sick headache. The attack began early in the morning, getting worse as the day advanced. I saw her at 3:00 P. M., suffering intense pain in the head and down the spine. She said she felt as if a cord extended the whole length of the spine and was becoming too short, so that her head was drawn backward with each paroxysm of suffering. The pain is very severe all the time, but every few minutes a paroxysm of intense suffering comes on which almost induces convulsions. During the paroxysm the head is drawn backward, the thumbs drawn across the palms and the whole arm rotated inward, pupils dilated. As the stomach seemed to be somewhat distended, I gave an emetic, ten grains of powdered ipecac in a tumbler full of warm water. After half an hour, there being no nausea, and the pain appearing to be more severe, ten grains more of ipecac with the addition of a teaspoonful of bicarbonate of soda was given. Waiting on this fifteen minutes, there still being no nausea, and thinking that an irritant emetic might be more prompt, I gave her a teaspoonful of ground mustard in a tumbler full of warm water. After again waiting 15 minutes, with no nausea, the patient's suffering increased to such a degree that convulsions seemed imminent. I decided to use and gave one-tenth grain apo-morphine mur subcutaneously. Free emesis occurred in about five minutes, with immediate subsidence of all the trouble. The patient vomited what had been eaten at dinner the day before. I have treated this patient in several similar attacks since, giving apo-morphine at once with prompt relief.

Case 2. Mr. S., Irish, aged about 45, a hard drinker, in January, 1890, took by mistake, or with suicidal intent, about one and one-half ounce of vinegar, in which he had previously dissolved some strychnine. He claimed to have taken about half the contents of the bottle. One and one-half ounces remained in the bottle. I could not ascertain exactly the amount of strychnine dissolved in the vinegar, however, he used all that he procured from the druggist. The strychnine was purchased several months previous and the druggist who furnished him lost his poison book by fire, but thought the amount was either three or five grains. Mr. C.'s family were not at home when he took the strychnine and it is not known at what hour he took it or at what time the first symptoms were manifested. I saw him at 6:00 P. M. A physician who happened to be passing had been called in and had given him a hypodermic injection of morphine, one-quarters grain atropine, one one-hundred and fiftieth grain about fifteen minutes before my arrival. Having treated this patient on a former occasion for an attack of suspended animation which I thought largely due to some gastric derangement and thinking that this might be a similar case with the variation, I at once gave subcutaneously 1-15th grain apo-moiphine mur, which was followed in five minutes by ineffectual efforts at vomiting and complete cessation of the spasms which had been recurring at very short intervals, the patient semi-conscious during the attack, partially asphyxiated. The patient had but one spasm after my arrival before he was relieved by apo-morphine. Still thinking that there must be something in the stomach which caused the trouble, I decided to use a stomach pump, which I sent for. At 7:30 P. M., with the assistance of Dr. G. H. Winston, I made several unsuccessful attempts to pass the tube, spasm of the parts preventing. The patient now began to have recurrence of spasms, the character of which led me to suspect strychnine poisoning. While recurring at short intervals, the spasms could be provoked by sudden noise or by slapping him. Morphine and atrophia were again administered subcutaneously but after waiting a few minutes and seeing no abatement of the symptoms, at 8:00 P. M. he was given 1-10th grain apo-morphine. Spasms ceased at once; some nausea, but no effort to vomit followed the dose. Patient's urine was now drawn with a catheter and set aside with a view to testing for strychnine. The patient was given two oz. castor oil and the dose repeated after an hour. At 11:00 P. M. he was still resting quietly. At 12:00 o'clock the spasms recurred, of a character more severe than before and death by asphyxia seemed imminent. Apo-morphine, grain ⅛ was given, which at once arrested the spasms, but caused no attempt at vomiting. He did not complain of nausea. At 1:00 A. M. he was resting well. Very soon the patient asked for some melted hog's lard, of which he drank a teacupful. Very soon thereafter his bowels acted freely. He rested well the remainder of the night. There was no recurrence of spasm after 12:00 o'clock. Dr. G. H. Winston tested the urine, drawn with the catheter and found unmistakable evidence of strychnine. Two days later, patient made confession of taking strychnine and gave me the bottle containing the residue. This solution was also tested and found to contain strychnine. The patient has since committed suicide by the pistol route, making it probable that suicide was intended before.

Case 3.—Mrs. ----, aged 20, mother of one child, about 18 months old, has not been in good health since the birth of her child. Was called to see her in June, 1890, on account of a violent attack of palpitation of the heart, with great dyspnoea. She had had several attacks of this character before. She was given subcutaneously 1-20th grain apo-morphine, vomiting of undigested food followed in five minutes, together with the relief of her distress.

Case 4.—Mr. J. T., aged 23, had an aching tooth, took two drinks of mean whiskey and repaired to the dentist where his tooth was extracted. He returned home five miles in the country very much intoxicated. At 6:00 P. M. he put his head into a tub of cool water and fell over with a convulsion which his excited friends said lasted fully fifteen minutes before he breathed freely. The spasms were repeated at short intervals until I saw him at 11:00 P. M. He was at once given subcutaneously 1-20th grain apo-morphine. The spasms ceased and did not return. Slight nausea, but no emesis followed this dose.

Other cases I could mention illustrative of the beneficial effects of apo-morphine, but I think the cases cited sufficient to present this new use of the remedy, that of preventing and controlling convulsions and other motor disturbances. I have seen so little written on the uses of apo-morphine, and that usually in relation of its action as an emetic, that I conclude that it is not appreciated at its full value by the profession. The works on materia medica and therapeutics in my collection—Brunton, Bartholow, Potter and Trosseau, while noticing other uses of apo-morphine, treat of it principally in relation to its action as an emetic.

I have not been able to find any report of the use of apo-morphine in motor disturbances for other than the relief to be expected from emptying the stomach, except the case in which a German physician succeeded in cutting short epileptic attacks by the subcutaneous injection of apo-morphine during the aura. See Med. Record 1884, page 598. Dr. Edward Cockrell, of England, is reported to have successfully treated infantile convulsions by its use. See Med. Record May, 1884, page 544. It is not stated whether the convulsions resulted from gastric disturbances or not, but as this is the usual cause of convulsions in children, we may suppose that its emetic action was sought in this case. I think it will be found that the remedy has heretofore been used principally for its emetic action. That certainly was my object in using it, until I accidentally found that it controlled the spasms produced by strychnine poisoning. Since that time I have used apo-morphine for all convulsions and in no case have I yet been disappointed as to the results.

Of the action of apo-morphine as an emetic, expectorant, or local anaesthetic, it is not my purpose to treat in this article, but to especially call attention to the fact here given that it does arrest or prevent spasms or other motor disturbances. From the results obtained from its use in my limited experience, I would confidently expect it to control any spasm not caused by lesion of the brain or spinal cord. I am not yet prepared to defend any special theory to account for the relief afforded in these cases, but I think that its depressive action on the motor centres, at the same time that it excites the vomiting centre, may relieve general convulsions by substituting a partial, namely vomiting, but whether we are able to satisfactorily explain the modus operandi or not, we have the facts which sustain the assertion that it does arrest spasm when present, and prevent it when about to occur. It is quite probable that this remedy will be of service in controlling the spasm during an attack of tetanus or rabies. I have had no opportunity of testing its value in either case, but from the analogy existing between these affections and poisoning by strychnine, I think we might reasonably give it a trial in these affections. I have used the remedy in quite a number of minor hysterical phenomena and one case of convulsions of that character with prompt relief. I have had no case of puerperal eclampsia since I began using this remedy and am not able to report its effect in that dreaded affection, though its use has arrested symptoms which led me to expect an attack.

My use of apo-morphine has been in doses ranging from 1/8 to 1-20th grain and has been uniformly by subcutaneous injection, except in one case, as an expectorant, with not better results than might be expected from other remedies of that class. I have in no case witnessed alarming symptoms from its administration. That its use is attended with danger in certain cases is too well attested to require comment.

